# Initial Post-Release Performance of Cultured *Cyprinus chilia* Juveniles in a Shallow Lake in Southwestern China

**DOI:** 10.3390/ani13203196

**Published:** 2023-10-13

**Authors:** Tingbing Zhu, Deguo Yang, Jinling Gong, Chunyong Wang, Xiangjun Miao, Yongben Liang, Xuemei Li

**Affiliations:** 1 Key Laboratory of Freshwater Biodiversity Conservation, Ministry of Agriculture and Rural Affairs, Yangtze River Fisheries Research Institute, Chinese Academy of Fisheries Science, Wuhan 430223, China; zhutb@yfi.ac.cn (T.Z.); yangdg@yfi.ac.cn (D.Y.); jlgong123@163.com (J.G.); 2 Fishery Workstation of Tonghai County, Yuxi 652799, China; wang62018@126.com; 3 Yunnan Institute of Fishery Sciences Research, Kunming 650051, China; kunmingmiao@126.com; 4 Fishery Workstation of Yuxi, Yuxi 653101, China; lyblyben@163.com

**Keywords:** mark–release–recapture, *Cyprinus chilia*, post-release performance, movement, gut microbiota

## Abstract

**Simple Summary:**

The post-release performance of cultured fish is crucial for evaluating the effectiveness of stock enhancement programs. Through a mark–release–recapture experiment, we studied the initial post-release performance characteristics of cultured *Cyprinus chilia* juveniles after being released into a shallow lake in southwestern China. Most of the released fish preferred to inhabit the lake shore zone with water depth less than 300 cm. The majority of long-distance movements (greater than 100 m) occurred within the first 4 days after release. The gut fullness of the released fish showed a trend of initially decreasing and then increasing, and the gut microbial community structure was significantly different from before release. This study suggests that cultured *Cyprinus chilia* juveniles can primarily adapt to the wild environment after 4–5 days of release.

**Abstract:**

The post-release performance of cultured fish is crucial for understanding the viability of cultured fish and assessing the effects of stock enhancement programs. This study aimed to investigate the initial post-release performance of cultured *Cyprinus chilia* juveniles by examining their movement, spatial distribution, gut fullness, and gut microbiota in nature. In July 2022, a total of 20,000 *C. chilia* juveniles, tagged with visible implant fluorescence (VIE), were released into Qilu Lake, a shallow lake in southwestern China. Subsequently, continuous recapture was conducted at fixed recapture sites using trap nets during the first 7 days, one month and three months after release. Out of the released fish, 512 were recaptured, resulting in a recapture rate of 2.56%. The recaptured fish had a 100% tag retention rate. The majority (98.05%) of the recaptured fish were found in the recapture sites located on the eastern or western lakeshore, while only 10 fish were recaptured from the recapture sites in the northern lake area. The water depth range where the recaptured fish were found ranged from 190 to 350 cm, with most fish preferring depths less than 300 cm. The majority of the released fish migrated towards the eastern and western lakeshore, with long-distance movement (greater than 100 m) primarily occurring within the first four days after release. The level of gut fullness in the released fish initially decreased and then increased over time following release. Regarding gut microbiota, the dominant phyla observed in most samples were Firmicutes, Proteobacteria, Cyanobacteria, and Fusobacteria. Furthermore, significant variations in the dominant genera were observed across different samples. Principal coordinates analysis (PCoA) revealed clear separation between the microbial communities of pre-release and post-release *C. chilia* juveniles. This study demonstrated that VIE tagging was a suitable method for short-term marking of *C. chilia* juveniles. Lakeshores with water depths less than 300 cm were identified as preferred habitats for *C. chilia* juveniles. The primary adaptation period for cultured *C. chilia* juveniles released into the natural environment was found to be approximately 4–5 days. These findings contribute to our understanding of the post-release performance of cultured fish and may provide guidance for the management and evaluation of relevant stock enhancement programs.

## 1. Introduction

The effectiveness of stock enhancement programs is significantly influenced by the post-release performance of cultured fish. While large-scale stock enhancements are commonly conducted worldwide, successful cases have been limited [1]. One of the reasons for this failure is the insufficient understanding of the biology and ecology of the stocks targeted for enhancement [2]. Additionally, studies on the performance and behavior of fish stocks have been scarce and more challenging compared to research on their general biological characteristics such as growth, food habits, and reproduction. Previous reports on the post-release performance of fish have primarily focused on adult or large-sized fish species, such as *Acipenser sturio*, *Argyrosomus japonicus*, *Anguilla japonica*, *Etelis coruscans*, and *Myxocyprinus asiaticus* [3,4,5,6,7]. In China, numerous stock enhancement programs have been implemented in recent decades, with their scale expanding each year. However, most of these programs in China have targeted small-sized juvenile cultured fish, highlighting the need for further research on the post-release performance of small-sized cultured fish.

There is currently no recognized standard for what indicators to use and how long the research period should be in order to accurately evaluate the performance of released fishes. There are various indicators utilized to assess the post-release performance of cultured fishes, although they lack a unified approach. Previous research has employed indicators such as growth [8,9], survival [10], dispersal or behavior [5,6], and physiology [11] to evaluate post-release performance. In addition, conducting a long-term recapture study is challenging and labor-intensive. Typically, the initial stage of release is of utmost importance in determining the viability of cultured fish, as the majority of mortalities occur within a short timeframe following their release [12]. Furthermore, monitoring and assessing their initial performance is considerably more feasible.

Mark–release–recapture is the fundamental method used to study the post-release performance of artificially released populations. Common fish tagging methods include listing, visible implant fluorescence (VIE), coded wire tags (CWT), and acoustic telemetry. The selection of appropriate tagging methods should be based on factors such as fish species, fish size, tagging cost, and characteristics of the study area. Due to its advantages of individual recognition and no need to recapture, acoustic telemetry was widely used in studies about the post-release movement of fishes, such as applications on *Acipenser sturio* [3], *Argyrosomus japonicus* [4], *Anguilla japonica* [5], *Etelis coruscans* [6], and *Myxocyprinus asiaticus* [7]. However, the acoustic telemetry was expensive and had large tags, which made it unsuitable for small-sized juvenile fish. Alternatively, VIE and CWT were recommended to the mark–release–recapture studies of small-sized fish as they were low cost and did minimal harm. For example, Leber et al. [13] reported on the recruitment patterns of juvenile, cultured Pacific threadfin (*Polydactylus sexfilis*) from the Polynemidae family. The study released the fish along sandy marine shores in Hawaii using CWT. Hervas et al. [9] also revealed the growth, survival, and distribution of released *Atractoscion nobilis* using the CWT method. The initial post-release movement of cultured *Cyprinus pellegrini* juveniles was evaluated using the VIE method [14].

*Cyprinus chilia* is endemic to the plateau lakes in Yunnan Province, China [15]. *Cyprinus chilia* is omnivorous and mainly feeds on large benthic invertebrates. Under natural conditions, *C. chilia* can grow up to 4 kg and is an important economic fish [15]. However, the natural resource of *C. chilia* has declined rapidly over the last decades due to overfishing, deterioration of the water environment, habitat loss, invasion of alien species, and other factors. At present, it is difficult to find wild populations of *C. chilia* in its naturally distributed lakes. To protect this endangered species, artificial breeding [16] and stock enhancement of *C. chilia* have been successfully implemented. However, there is a lack of information regarding the post-release performance and evaluation of these stock enhancement efforts. Also, a suitable tagging method for *C. chilia* juveniles has not been evaluated yet. Therefore, the management and planning of *C. chilia* stock enhancement programs remain unclear.

VIE was one of the most popular tagging methods used to mark released fish in China, but there are few reports about the effects of VIE tagging on the assessment of stock enhancement. Therefore, the objectives of this study are (1) to assess the effectiveness of VIE as a short-term tagging method for *C. chilia* juveniles and (2) to investigate the initial post-release performance of cultured *C. chilia* juveniles by monitoring their movement, spatial distribution, gut fullness, and gut microbiota. Correspondingly, two hypotheses are proposed: (1) the VIE should be suitable for the short-term tagging of *C. chilia* juveniles, and (2) cultured *C. chilia* juveniles should go through an obvious transitional period in the new environment. The findings of this study will contribute to a better understanding of the post-release performance of cultured Cyprinus fishes and will be valuable for the management and evaluation of stock enhancement programs related to *C. chilia*.

## 2. Materials and Methods

### 2.1. Fish

The fish were artificially hatched *C. chilia* juveniles produced at the Raofu fish farm, Tonghai County. Before the mark–release–recapture trial, fish were about 4 months old and had a mean total length (*L*) of 10.8 ± 1.2 cm (mean ± standard deviation, *n* = 46) and mean body weight (*W*) of 17.54 ± 5.19 g.

### 2.2. Mark–Release–Recapture Trial

A mark–release–recapture trial was conducted to examine the movement, spatial distribution, gut fullness, and gut microbiota of *C. chilia* juveniles after release. VIE tags were used in the study as its low cost and minimal harm to small-sized fishes.

A total of 20,000 *C. chilia* juveniles were selected for the mark–release–recapture trial. All of the selected fish were tagged with VIE tags to help the identification of fish samples in the subsequent recapture study. The tagging work was carried out on 25–26 July 2022. Prior to the tagging operation, fish were anesthetized using 30 mg/L of MS-222 for 1~2 min. VIE tagging procedures were as follows: red VIEs were injected into the epidermis of the head skin using hand-pressurized syringes (Northwest Marine Technology, Anacortes, WA, USA). The presence of the tags was visually confirmed. Following the tagging process, the fish were transferred to clean water to facilitate their recovery (recovery rate 100%).

**Figure 1:**
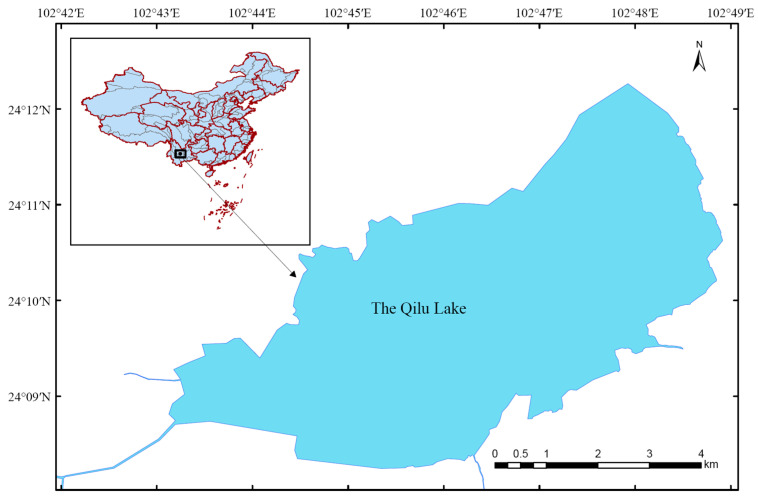
Geographic location diagram of Qilu Lake.

The designated water area for release is the Qilu Lake in Yunnan Province, China (Figure 1), as it is one of the main distribution lakes of *C. chilia*. The Qilu Lake has a maximum length of 10.4 km, maximum width of 4.4 km, water area of 38.86 km^2^, a maximum water depth of 540 cm, and an average water depth of 403 cm. There were 11 species of fish historically distributed in Qilu Lake, including *C. chilia*, *Cyprinus pellegrini*, *Cyprinus ilishaestomus*, *Cyprinus yunnanensis*, *Anabarilius qiluensis*, *Carassius auratus*, *Misgurnus anguillicaudatus*, *Oryzias latipes*, *Channa argus*, *Monopterus albus*, *Silurus grahami* [17]. There has been no record of *C. chilia* in Qilu Lake since 2000s. According to our investigation of the entire Qilu Lake on 12–14 July 2022, the present fish species include *Cyprinus carpio*, *Carassius auratus*, *Abbottina rivularis*, *Hemicculter Leuciclus*, *Pseudorasbora parva*, *Cultrichthys erythropterus*, *Pelteobagrus fulvidraco*, *Misgurnus anguillicaudatus*, *Paramisgurnus dabryanus*, *Rhinogobius giurinus*, *Hyporhamphus intermedius*. The release site was situated on the south shore of Qilu Lake, with a water depth of 220 cm, transparency of 37 cm, water temperature of 27.4 °C, dissolved oxygen level of 10.46 mg/L, a pH of 8.83, and conductivity of 891 μs/cm. On 27 July 2022, the tagged fish were packed into double-layered nylon fish bags (80 cm × 40 cm) with a density of 150~200 fish per bag. The volume ratio of oxygen to water in the bag was approximately 3:1. The fish bags were then transported (approximately 0.5 h) to the release site for release. The entire release process was conducted manually. The fish bags were initially submerged in the water at the lakeshore to balance the temperature for 0.5 h. Afterward, the fish bags were untied, and the tagged fish were carefully released into the lake.

The recapture work was divided into two stages, including a 7-day continuous recapture and a long-term recapture.

**Figure 2:**
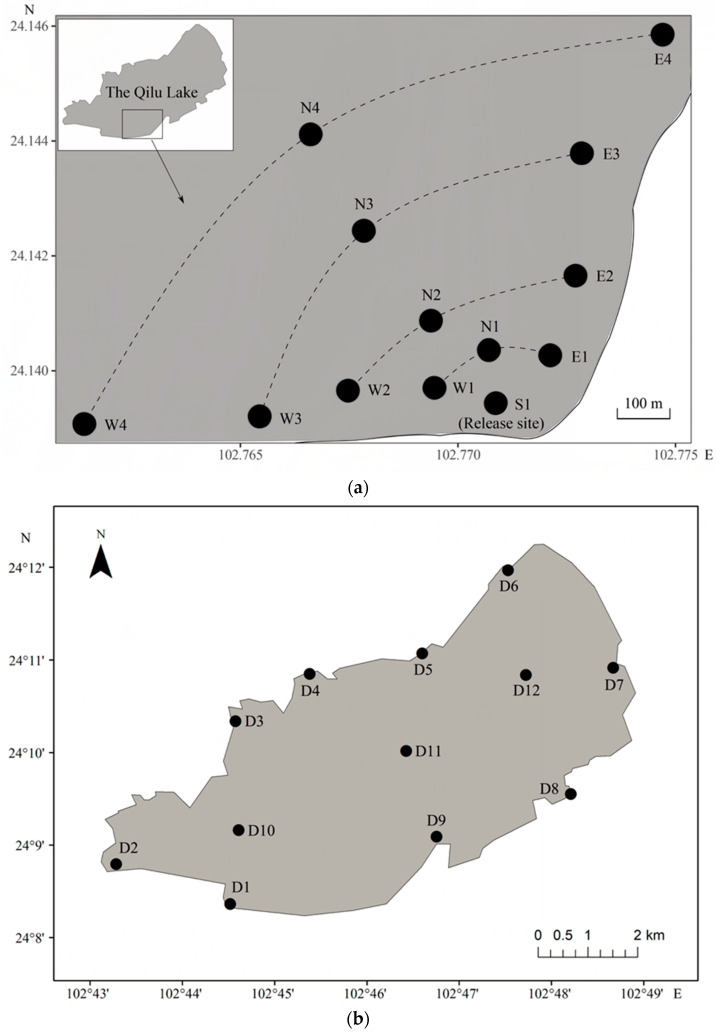
Recapture sites of cultured *Cyprinus chilia* juveniles released into Qilu Lake. (**a**) Sites in key recapture area; (**b**) sites in regular recapture area.

The 7-day continuous recapture was conducted immediately following the release of fish. The lake was divided into key recapture area and regular recapture area. The key recapture area was around the release site (Figure 2a). A total of 13 fixed recapture sites were set up in the key recapture area, including 1 site (S1) at the release site, 4 sites (E1, E2, E3, E4) on the eastern lakeshore, 4 sites (W1, W2, W3, W4) on the western lakeshore, and 4 sites (N1, N2, N3, N4) in the northern lake area (Figure 2a). This created a 4-layer arc recapture monitoring network, with the release point as the origin. The distances from the recapture sites on the 1st, 2nd, 3rd, and 4th layers to the release site were set as 150, 300, 500, and 800 m, respectively. However, due to limitations in terrain, interference from wind and waves on the positioning ship, and other factors, there was some variation between the actual distance between adjacent recapture sites and the preset values (Table 1 and Table 2). The regular recapture area included lake areas outside of key recapture area (Figure 2b). A total of 12 fixed recapture sites were set up in the regular recapture area, including sites D1, D2, D3, D4, D5, D6, D7, D8, D9, D10, D11, and D12 (Figure 2b).

The long term recapture was conducted one month and three months after release. The recapture sites for the long term recapture was the same as the recapture sites in the regular recapture area of the 7-day continuous recapture, plus the release sites (S1). For each long-term recapture, 3-day continuous recapture was conducted.

Recapture gears used were trap nets with dimensions of 10 m in length, 45 cm in width, 33 cm in height, and a mesh size of 7 mm. The maximum volume of the net was 1.485 m^3^. A density of one net per site was set, and the nets were deployed 2 h prior to fish release. After the fish were released, the catch in the nets was collected every 24 h, and the nets were then repositioned during the recapture periods. It should be noted that the net at S1 had a water entry time of only 6 h on the fifth day after release, as it was affected by an incorrect removal of the net.

### 2.3. Processing of the Recaptured Fish Samples

All recaptured fish samples were identified by species. For *C. chilia* juveniles, the tag presence of fish was determined through visual examination. The recaptured *C. chilia* juveniles were counted separately based on the date and recapture site. When fewer than 30 released fish samples were recaptured at a site per day, the total length and weight of all fish samples were measured. If more than 30 released fish were recaptured at a site per day, a random sample of 30 fish was measured for total length (accurate to 0.1 cm) and weight (accurate to 0.1 g).

According to the number of fish samples, 3–20 fish were randomly selected for dissection every day to assess the feeding intensity of the released fish by examining their gut fullness levels [18]. The level of gut fullness ranged from 0 to 5, representing feed intake from low to high (Table 3). Additionally, freshly dissected guts of three fish samples were collected every day for analysis of gut microbiota structure. According to the post-release days and sampling order, the samples were named as D0–1, D0–2, D0–3, D1–1, D1–2, D1–3, D2–1, D2–2, D2–3, D3–1, D3–2, D3–3, D4–1, D4–2, D4–3, D5–1, D5–2, D5–3, D6–1, D6–2, D6–3, D7–1, D7–2, and D7–3, respectively. After sampling, any remaining surviving fish were transferred to indoor breeding tanks for temporary cultivation.

### 2.4. Gut Microbiota Structure Analysis

Total microbiota genomic DNA was extracted from approximately 0.3 g of the freshly dissected intestine of each fish using the FastDNA spin kit for soil (MP, Solon, OH, USA) following the manufacturer’s protocols. The 515F (5′-GTGYCAGCMGCCGCGGTA-3′) and 907R (5′-CCGTCAATTCMTTTRAGT-3′) primers [19,20] were used to amplify the bacterial 16S rRNA gene V4–V5 fragments. PCR integration and protocols were carried out as follows: 94 °C for 3 min followed by 30 cycles of 94 °C for 40 s, 56 °C for 60 s, 72 °C for 60 s, and a final extension at 72 °C for 10 min until the reaction was halted by the user. The amplicons were purified and sequenced using the Illumina Miseq platform at Guangdong Meilikang Bio-Science Ltd. (Foshan, China).

Raw reads were merged using FLASH 1.2.8 and subsequently processed using QIIME 1.9.0, as described [21]. Briefly, all the merged sequences were assigned to each sample based on their barcode sequences, and the trimmed barcodes and primer sequences were removed using QIIME 1.9.0 software. Low-quality and chimeric sequences were removed using QIIME 1.9.0 and UCHIME 4.2.40, respectively. Subsequently, the remaining high-quality sequences were clustered into operational taxonomic units (OTUs) with 97% identity using UPARSE 7.1 [22]. The taxonomy of each OTU was assigned using the RDP classifier [23] in the gg_13_8 database. Alpha diversity indices were calculated using QIIME version 1.9.0. The Illumina sequencing raw data obtained from this study were deposited in the NCBI Sequence Read Archive with accession No. PRJNA999371.

### 2.5. Data Analysis

The condition factor, recapture rate, tag retention rate, fish distribution density, water depth selectivity, and daily movement velocity were calculated.

Condition factor:

*CF* = 100% × *W*/*L*^3^

where *CF* was condition factor, *W* was body weight (g), and *L* was total length (cm).

Recapture rate:

*C* = 100% × *n*/*N*

where *C* was recapture rate, *n* was the number of recaptured releasing fish, and *N* was the number of releasing fish.

Tag retention rate:

*R* = 100% × *n_p_*/*n*

where *R* was the tag retention rate, *n_p_* was the number of recaptured *C. chilia* with VIE tag, and *n* was the total number of recaptured *C. chilia*.

Fish density:

*D_i_* = *N_i_*/*V_i_*

where *D_i_* was the density (ind./m^3^) of fish at recapture site *i*, *N_i_* was the number of recaptured releasing fish per day at recapture site *i*, and *V_i_* was the fully extended volume of the trap net used at recapture site *i*.

To understand any preference of released fish for water depth, a water depth selectivity index was calculated. The recapture sites were divided into 4 water depth groups based on their respective water depths (≤250 cm, 251–350 cm, 351–450 cm, ≥451 cm). The water depth selectivity index was calculated using the following function:

$$S_{j}=N_{j}/\sum_{j}^{m}N_{j}$$

where *S_j_* was the selectivity of the releasing fish for the water depth group *j*, *N_j_* was the mean number of recaptured fish at the recapture sites from the water depth group *j*, *m* was the number of water depth groups. The range of water depth selectivity values is 0~1 (0 means complete avoidance, 1 means total selectivity).

The daily movement velocity of the released fish in different directions (east, west, and north) in the first 7 days after release was calculated to assess the movement pattern of releasing fish using the following function:

$$V=\sum L_{i}N_{ij}/(T_{i}\sum N_{ij})/(T_{j}\sum N_{ij})$$

where *V* was mean daily movement velocity of the releasing fish towards a certain direction, *L_i_* was the linear distance from the release site (S1) to site *i* in a certain direction, *N_ij_* was the number of recaptured releasing fish at recapture site *i* on day *j*, and *T_j_* was the number of days post release.

The α-diversity indices of the gut microbiota were determined using the richness index, Shannon index, Simpson index, and Chao1 index of observed OTUs in each sample.

The difference in individual size and α-diversity indexes of the gut microbiota among groups was compared using one-way ANOVA. A correlation heatmap of dominant microbial genera was analyzed using the corrplot R package. Beta diversity was calculated through unconstrained principal coordinate analysis (PCoA) based on weighted UniFrac distance to display the differences in gut microbial community structure among different sampling days. A significant difference was set at a value of *p* < 0.05.

## 3. Results

### 3.1. Recapture Rate

Only the 7-day continuous recapture was successful in collecting the released fish, and all of the recaptured samples were from the key recapture area.

**Figure 3:**
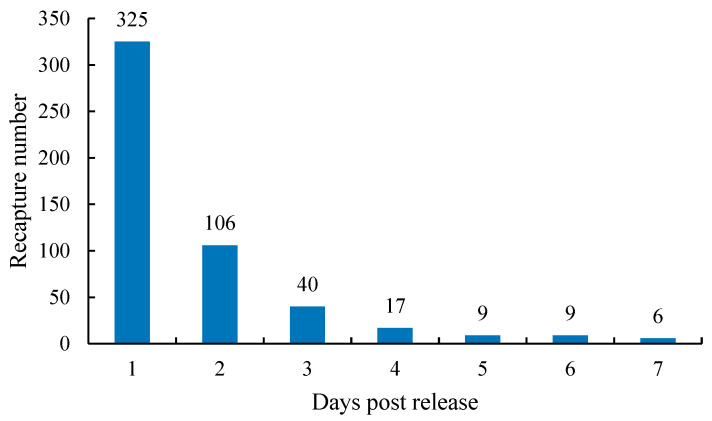
Daily recapture number of cultured *Cyprinus chilia* juveniles in Qilu Lake post-release.

A total of 512 fish were recaptured over the 7 day period, resulting in a recapture rate of 2.56%. The number of recaptured fish decreased as the time post-release increased (Figure 3). The total length, body weight, and condition factors of the recaptured fish were generally smaller than those randomly measured pre-released samples (Table 4).

### 3.2. Tag Retention Rate

**Figure 4:**
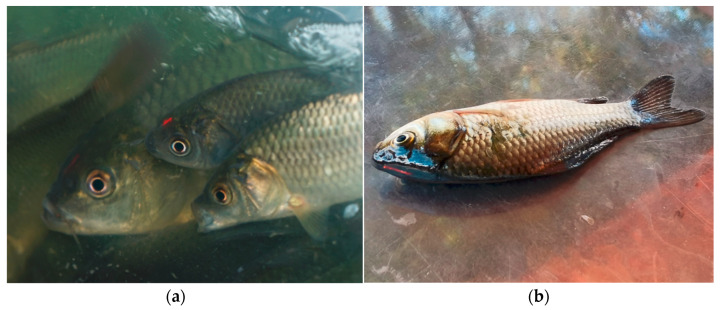
VIE-tagged cultured *Cyprinus chilia* juveniles before release (**a**) and post-release (**b**) into Qilu Lake.

All recaptured fish were identified using VIE tags (Figure 4) with a tag retention rate of 100%. Furthermore, no obvious injuries or illnesses were found in any of the samples, indicating that the VIE tagging operation had limited effect on the short term survival of the released fish.

### 3.3. Post-Release Movement Patterns

**Figure 5:**
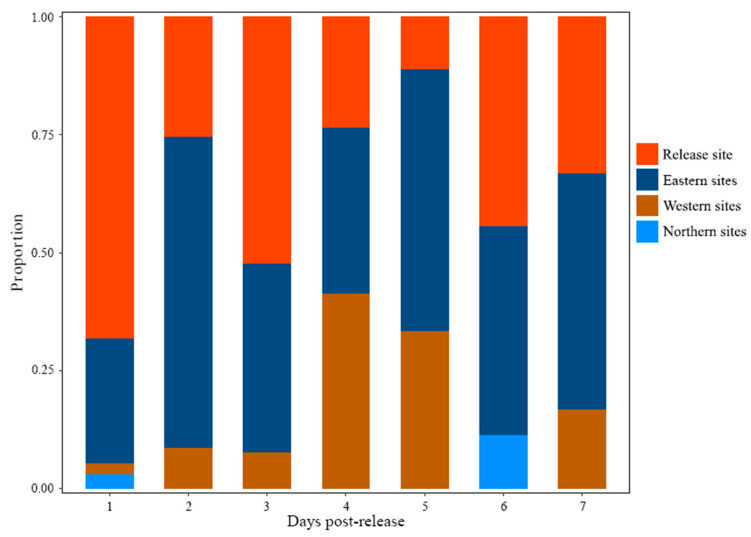
Relative proportion of recaptured cultured *Cyprinus chilia* juveniles from different direction sites post-release into Qilu Lake.

The post-release movement patterns of cultured *C. chilia* juveniles were analyzed by examining the recaptured fish numbers from various directions (Figure 5).

**Figure 6:**
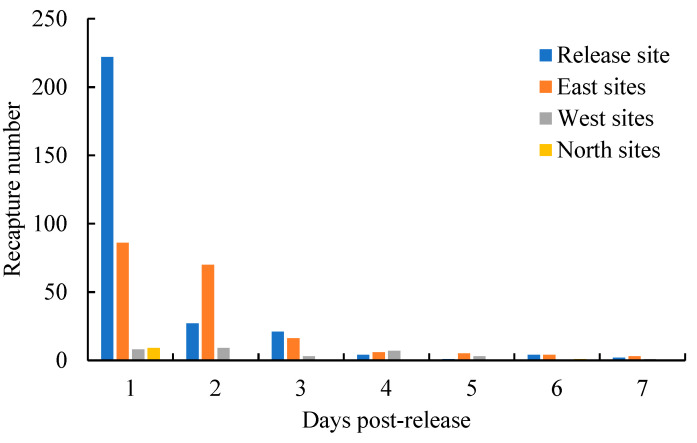
Number of recaptured cultured *Cyprinus chilia* juveniles from different direction sites post-release into Qilu Lake.

On the first day after release, the majority of the fish remained near the release site. Some fish had moved to the eastern lakeshore, while a few others headed towards the western lakeshore and the northern area of the lake. During the second day to the seventh day after release, the relative proportion of fish moving towards the eastern and western lakeshore gradually increased. Notably, the proportion of fish moving towards the eastern lakeshore was higher than that of the western lakeshore. The proportion of fish moving towards the northern lake area was relatively small, and the recaptured fish from sites in the northern lake area were only recorded on the 1st and 6th day after release. The movements away from the release site A total of 231 fish were recaptured at recapture sites excluding the release site, and 87.01% of them were recaptured within the first three days post-release (Figure 6), which indicated the majority of movements away from the release site occurred within the first three days post-release.

The individual size of fishes moving towards the eastern lakeshore and the northern lake area were significantly larger than those remained at the release site (*p* < 0.05). However, there was no significant size difference between fishes moving towards the western lakeshore and those that stayed at the release site (Table 5).

### 3.4. Spatial Distribution Patterns

**Figure 7:**
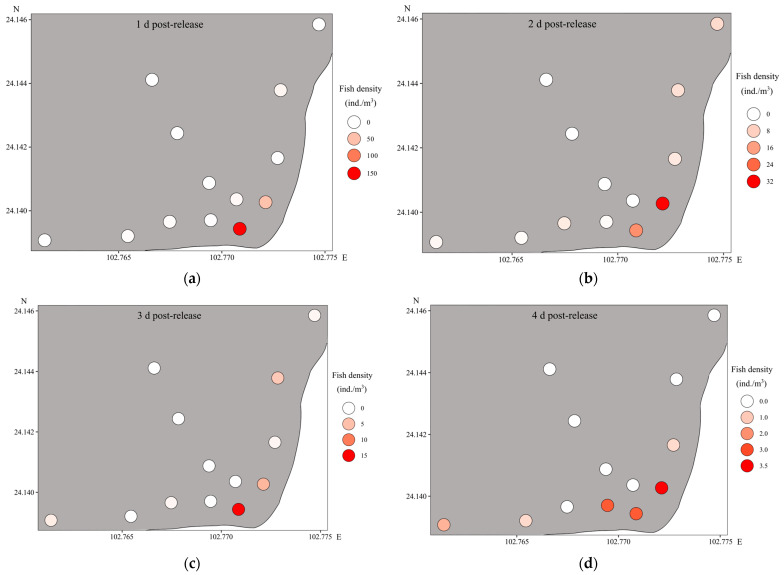

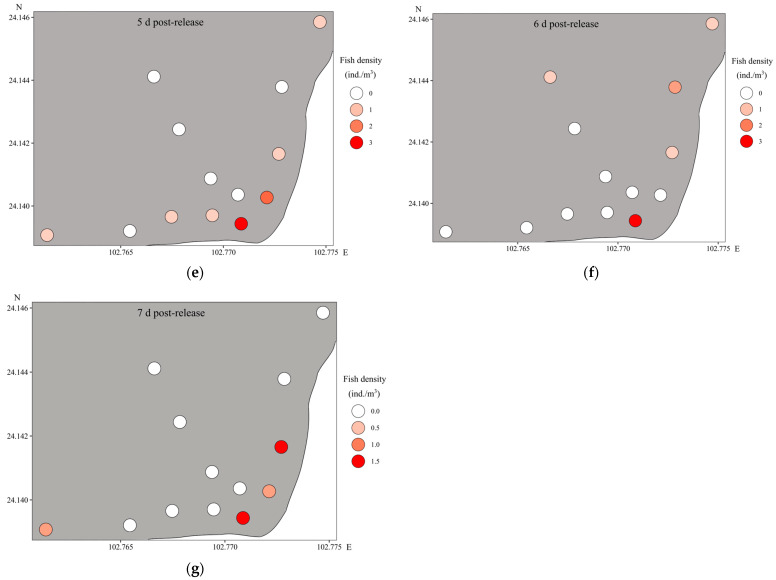
Spatial density dynamics of cultured *Cyprinus chilia* juveniles after being released into Qilu Lake: (**a**) 1 d post-release; (**b**) 2 d post-release; (**c**) 3 d post-release; (**d**) 4 d post-release; (**e**) 5 d post-release; (**f**) 6 d post-release; (**g**) 7 d post-release.

Upon release, fish initially formed large schools at the release site and in the vicinity. Subsequently, they gradually dispersed towards adjacent lakeshore regions (Figure 7).

### 3.5. Water Depth Selectivity

The recapture sites, where fish were successfully retrieved, exhibited a water depth range of 190–350 cm. It was observed that the majority of fish displayed a preference for water depths less than 300 cm (Table 6).

### 3.6. Movement Velocity

**Figure 8:**
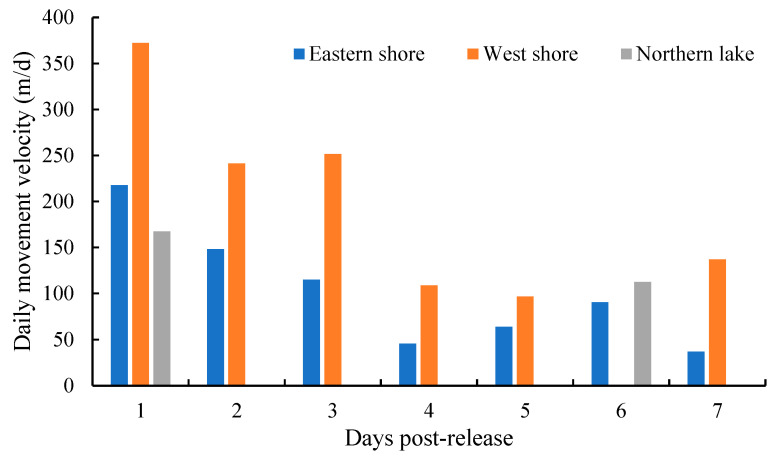
Daily movement velocity of cultured *Cyprinus chilia* juveniles in different directions post-release into Qilu Lake.

The movement velocity of the fish in the eastern and western lakeshore initially decreased and then increased during the 7-day recapture period, with changes occurring on the 4th day (Figure 8). Comparatively, the movement velocity of fish was higher on the eastern lakeshore than on the western lakeshore. An individual fish with the highest velocity (813 m/d) was recaptured at site E4 on the first day after being released. This fish had a total length of 11.6 cm and a body weight of 19.6 g. However, it was difficult to assess the movement velocity of the fish in the northern lake area due to the limited number of fish samples.

### 3.7. Gut Fullness

**Figure 9:**
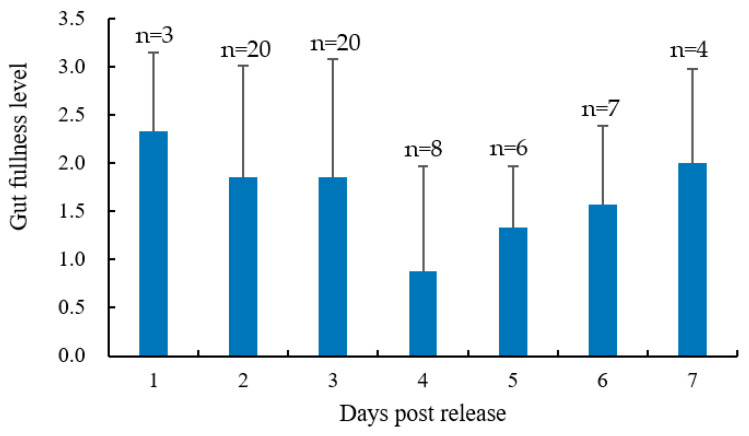
Gut fullness level dynamics of cultured *Cyprinus chilia* juveniles post-release into Qilu Lake. Error bars represent the standard deviation.

There was no significant difference in the levels of gut fullness among samples collected on different recapture days. However, released fish exhibited a pattern in the levels of gut fullness, when the average values were considered. Specifically, the level of gut fullness in the released fish decreased from day 1 to day 4 after release, and subsequently increased from day 4 to day 7 after release (Figure 9).

### 3.8. Gut Microbiota

**Figure 10:**
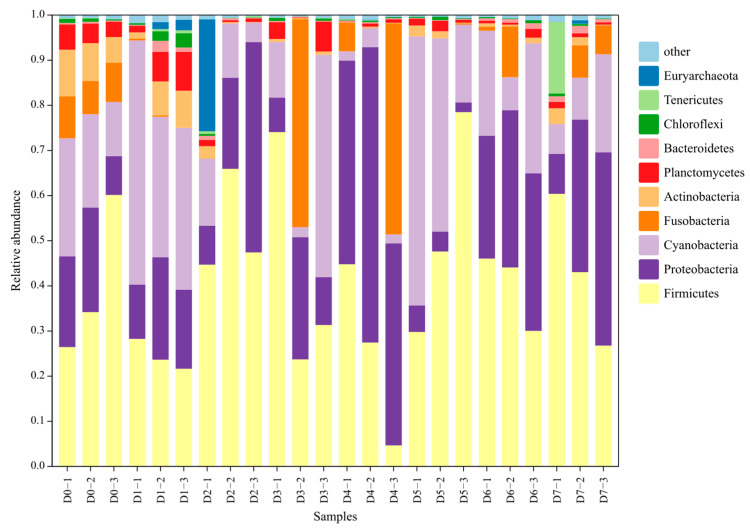
Relative abundance dynamics of dominant gut microbial phyla of cultured *Cyprinus chilia* juveniles post-release into Qilu Lake. D0–1 indicates the first sample of fish before release, D1–2 indicates the second sample of fish post-release for 1 day, etc.

After eliminating low-quality sequences, a total of 1,974,160 high-quality sequences were obtained from 24 samples of gut microbiota collected from *C. chilia* on different release days. To minimize the potential influence of varying sample sequencing depths on the subsequent analysis results, we randomly selected 30,485 sequences from each sample for further analysis. Among the detected sequences, a total of 56 phyla were identified. The dominant phyla in the gut microbiota were Firmicutes, Tenericutes, Proteobacteria, Cyanobacteria, Fusobacteria, Actinobacteria, Planctomycetes, Bacteroidetes, Chloroflexi, and Euryarchaeota. These dominant phyla accounted for 98.18 ± 0.05% of the high-quality sequences analyzed in this study (Figure 10).

**Figure 11:**
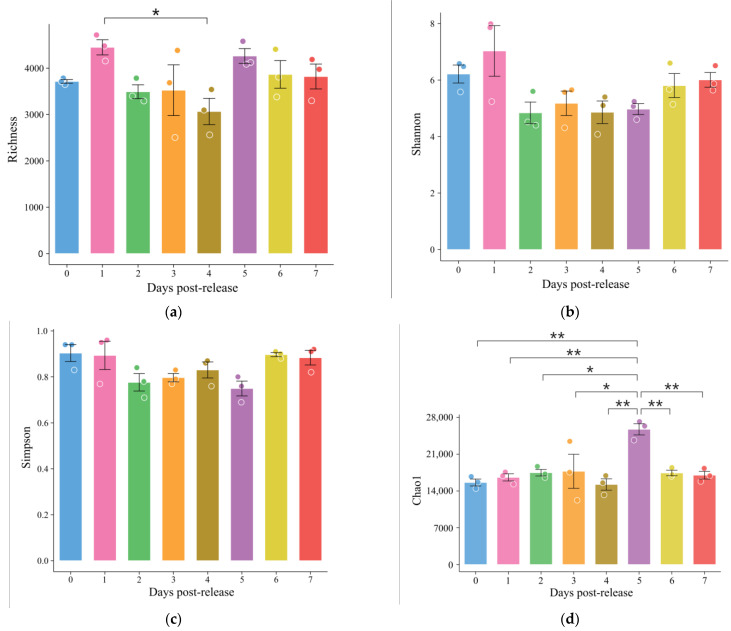
α-diversity index dynamics of the gut microbiota of cultured *Cyprinus chilia* juveniles post-release into Qilu Lake. (**a**) Richness index; (**b**) Shannon index; (**c**) Simpson index; (**d**) Chao1 index. Error bars represented the standard deviation. “*” indicate significant differences between groups (*p* < 0.05). Different colors represent different days post-release. “**” indicate extremely significant difference between groups (*p* < 0.01).

A total of 90,575 operational taxonomic units (OTUs) were identified. However, an average of 3773.96 ± 580.96 OTUs were detected in each sample, with a significant difference between the pre-release group and the group 4 days post-release (Figure 11a). The Shannon and Simpson indices of the gut microbiota showed no significant difference between the different groups. However, the Chao1 index of the 5-day post-release group was significantly different from the other groups (Figure 11b–d).

**Figure 12:**
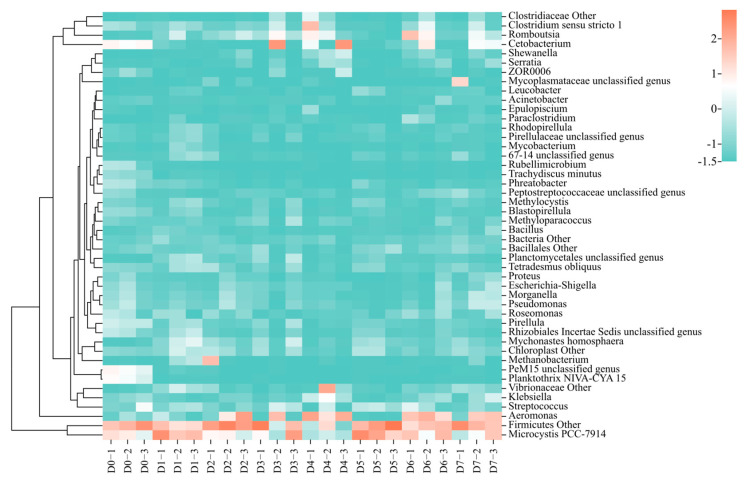
Heatmap profile of dominant gut microbial genera of cultured *Cyprinus chilia* juveniles post-release into Qilu Lake. D0–1 indicates the first sample of fish before releasing, D1–2 indicates the second sample of fish post-release for 1 day, etc.

At the genus level, a total of 2819 genera were detected in the gut microbiota, out of which 46 were found to be dominant (Figure 12). The dominant genera before the release were Firmicutes other, Microcystis PCC-7914, *Cetobacterium*, PeM15_unclassified genus, and Planktothrix NIVA-CYA 15. After the release, the dominant genera were Firmicutes other, Microcystis PCC-7914, *Aeromonas*, *Romboutsia*, and *Cetobacterium*.

**Figure 13:**
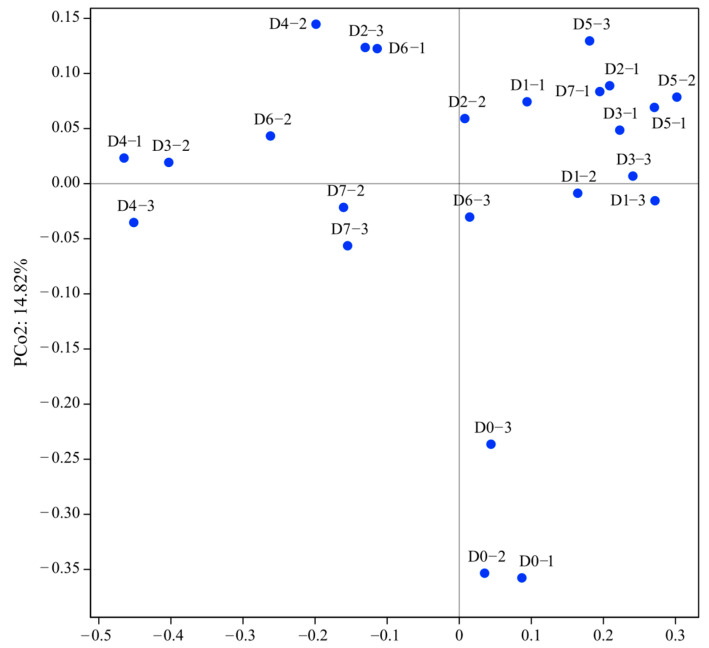
Principal coordinate analysis at the OTU levels of gut microbiota of cultured *Cyprinus chilia* juveniles post-release into Qilu Lake. D0–1 indicates the first sample of fish before releasing, D1–2 indicates the second sample of fish post-release for 1 day, etc.

The results of PCoA showed that the microbial communities in the D0 group were clearly separated from the other groups (Figure 13).

## 4. Discussion

Marking methods suitable for juvenile fish are crucial to improving the efficiency of behavioral studies on released fish and evaluating the effects of numerous ongoing stock enhancement programs. Previous studies on the post-release behaviors of fish have primarily relied on acoustic telemetry [4,5,6,7,24] and satellite archival tags [25]. These methods enable the monitoring of individual fish movement and habitat selection without the need for physical capture. However, these marking methods had large tags and were therefore unsuitable for smaller-sized juvenile fish. Furthermore, the cost associated with acoustic telemetry and satellite archival tags makes it impractical to tag a large number of fish. It is worth noting that electronic tags may also affect the short-term behavior of fish [26]. Consequently, marking methods such as VIE and CWT are preferred for mark–release–recapture studies of large quantities of small-sized fishes due to their affordability, minimal damage, ease of operation, and ease of identification. The findings of the present study demonstrate high VIE tag retention and minimal damage in all recaptured fish samples, indicating that VIE is a suitable marking method for short-term mark–release–recapture studies of *C. chilia*.

Many studies have confirmed that the hatchery environment can affect the post-release behavior of fishes [27]. On the first day after release, the majority of individuals remained near the release point, which is referred to as the “residency period” [4]. The localized behavior exhibited by the cultured *C. chilia* juveniles on the first day after release might be due to unfamiliarity in a new environment, or it could be an extension of the settlement behavior developed in the artificial rearing environment. Similarly, hatchery-reared Mulloway (*Argyrosomus japonicus*) [4] and *Micropterus cataractae* [28] also showed a period of minimal movement when released into new environments with varying durations. From the second day after release, the *C. chilia* juveniles began to increase their movement and exploration of the environment. This may reflect the tradeoff between avoiding predation and resource utilization in the new environment. As time passes, factors such as hunger may motivate *C. chilia* juveniles to leave the release point and spread to more unfamiliar waters in order to increase their opportunity to obtain resources.

The released fish showed significant habitat selectivity. As an important parameter of fish habitat selectivity, water depth preference were reported in many fish species. For example, *Acanthopagrus schlegelii* prefers water depths of 8–10 m, while *Lutjanus argentimaculatus* prefers a water depth of 1–3.5 m [29]. The movements of land-locked Atlantic salmon (*Salmo salar*) in a large lake were nearshore (<2 km from shore) from spring to summer at ~20 m bathymetric depths [30]. The present study found the released fish mainly remained in shallow waters near the release point. Selectivity of water depth showed that released fish preferred water depths of up to 300 cm. Furthermore, the movement of released fish towards the eastern lakeshore was greater in scale than that towards the western lakeshore. It is speculated that the difference in complexity of the lake shorelines in the east and west may be related. The shoreline near the western side of the lake was relatively straight, while the shoreline near the eastern side of the lake was winding and complex. This complex shoreline provides more diverse habitat conditions, which can be beneficial for fish by providing shelter and feeding opportunities. Other species, like *Acanthopagrus schlegelii*, also prefer to stay in areas where food is more abundant and easy to hide in the initial stages after release [31].

The post-release movement velocity of *C. chilia* juveniles fluctuated over the 7-day trial. The movement of *C. chilia* juveniles decreased over the first three days after release and remained low for the following four days. This suggests that the majority of movement by *C. chilia* juveniles occurs within the first three days after release. The dispersal or movement of different fish often varies after release. For example, the horizontal movement velocity of released deep-water longtail red snapper (*Etelis coruscans*) was 2.2 km/d [6], while the movement velocity of artificially bred Japanese eels (*Anguilla japonica*) after release into the sea can reach 2.31 km/h [5]. Lake trout (*Salvelinus namaycush*) released into Lake Ontario had an average swimming speed of 1.64 km/h over the first day [32]. Cyprinus fishes are generally sedentary. For example, the mean daily movements of grass carp (*Ctenopharyngodon idella*) in Lake Erie ranged from <0.01 to 2.49 km/d, and only 25% of studied grass carp had mean daily movements greater than 0.88 km/d [33]. Therefore, the movement of *C. chilia* juveniles in our study was also relatively slow. The maximum recorded movement velocity in the present study was 813 m/d, and the overall movement gradually decreased over time, which is similar to that of some other fish species. For example, the movement velocity of mature Chinese sucker (*Myxocyprinus asiaticus*) released into the Yangtze River also showed a declining trend over time [7]. The gradual adaptation to the new environment and the acquisition and retention of an ideal habitat space may be reasons for the declining daily movement distance of *C. chilia* juveniles in this study.

The levels of gut fullness in released *C. chilia* juveniles showed a trend of initially decreasing and then increasing, although no statistical differences were found among recapture days. Limited sample sizes may explain these results. However, the mean levels of gut fullness in the released fish decreased from day 1 to day 4 after release, and then increased from day 4 to day 7 after release. This suggests that the released fish may begin feeding on the 5th day after release. Based on changes in the movements of released fish, it may be inferred that the first four days post-release are a transitional period for *C. chilia* juveniles in adapting to the new environment. During this period, they need to find suitable habitats and adapt to consuming natural prey. By the fifth day after release, released fish have entered an adaptation period during which they have relatively stable habitats and feeding conditions. Similarly, the gut fullness of the hatchery-reared honmasu salmon *Oncorhynchus rhodurus* × *masou* parr, released a week prior, was found to be comparable to that of wild parr [11].

This study indicates that the structure of the gut microbial community in *C. chilia* juveniles undergoes significant changes after they were released. Our study provided the first report on the gut microbiota of *C. chilia*. From the perspective of the composition of gut microbial phyla, the variations within 7 days after release were not significant. However, there is a fluctuation in the dominant genus composition of gut microbes in *C. chilia* juveniles after release. The dominant genera of gut microbiota on the first day after release mainly included Firmicutes other, Microcystis PCC-7914, *Cetobacterium*, PeM15_unclassified genus, and Planktothrix NIVA-CYA 15. The members of Firmicutes and *Cetobacterium* perform digestion functions. For example, PeM15_unclassified genus is a member of the Actinobacteria phylum, which has been verified as an important group of PAOs (phosphorus-accumulating organisms) in enhanced biological phosphorus removal systems. Additionally, some members of this genus may contain nitrite reductase genes that are involved in denitrification [34]. Microcystis PCC-7914 and Planktothrix NIVA-CYA 15 are indicators of water body eutrophication, which can be used to assess the eutrophication levels in ponds used for fish rearing. Starting from the second day after release, the dominant genera continued to change, indicating that the *C. chilia* juveniles were undergoing a transitional period of adaptation to the new environment. Generally, the dominant genera after release were *Aeromonas* and *Romboutsia*. *Aeromonas* can secrete enzymes related to pathogenicity and environmental adaptation, such as hemolytic enterotoxin, lipase, protease, and amylase [35]. These enzymes improve the digestibility of food and play an important role in the digestion process. *Romboutsia* can ferment and metabolize macromolecular carbohydrates that are difficult for a host fish to digest into short-chain fatty acids, such as butyric acid. This process helps reduce intestinal pH, improve the host’s immune regulation ability, and maintain the balance of intestinal microecology [36]. The increase in these two genera may be an adaptive strategy of *C. chilia* juveniles to cope with the increased risk of food scarcity and stress infection after release.

Only the 7-day continuous recapture collected the released fish successfully, and the reasons for the failure to obtain samples over a longer period (1 month and 3 months after release) are unknown. We speculate that the dispersal of *C. chilia* after release may lead to a continuous decrease in density, thereby reducing the probability of catching released fish, or perhaps they experienced high post-release mortality. However, the present results may be sufficient to reveal the initial post-release performance of cultured *C. chilia* juveniles.

## 5. Conclusions

Our study highlights the initial post-release performance of cultured *C. chilia* juveniles and examines the applicability of VIE tag, water depth selectivity, feeding adaptation to natural diet, and gut microbiota of cultured *C. chilia* juveniles after release. Overall, the VIE tag was a suitable method for short-term marking of *C. chilia* juveniles. Both the results of behavioral and feeding indicated that an obvious transitional period of cultured *C. chilia* juveniles was 4–5 days post-release. However, there are still many remaining questions that need to be studied in future, such as the performance of released *C. chilia* in a longer term, reasons for the failure of recapture after *C. chilia* being released for more than 7 days, and the habitat selection mechanism of the released *C. chilia*.

Based on the present results, we propose the following suggestions: Firstly, stocking of *C. chilia* juveniles should be conducted in waters with diverse habitats and a depth not exceeding 300 cm. Secondly, due to their limited movement velocity, the release sites of *C. chilia* juveniles should be multiple (see example [37]) and dispersed to avoid intense local resource competition. Thirdly, natural bait should be acclimated prior to release to shorten the time it takes for *C. chilia* juveniles to resume feeding after release.

## Figures and Tables

**Table 1:** Water depth of the recapture sites and their distance to the release site in the mark–release–recapture trial of cultured *Cyprinus chilia* juveniles released into Qilu Lake.

Recapture Site	Water Depth (cm)	Distance to the Release Site (m)
S1	220	0
E1	200	157
E2	230	310
E3	200	524
E4	190	813
N1	350	104
N2	430	220
N3	490	454
N4	300	676
W1	300	145
W2	250	345
W3	250	551
W4	230	960
D1	160	2932
D2	200	5082
D3	120	4625
D4	180	4832
D5	250	5048
D6	160	7019
D7	100	6251
D8	150	3792
D9	190	1586
D10	395	3143
D11	540	3072
D12	403	5213

**Table 2:** Distances between the adjacent sites in the key recapture area of the mark–release–recapture trial of cultured *Cyprinus chilia* juveniles released into Qilu Lake.

Adjacent Recapture Site	Distance (m)
E1–E2	165
E2–E3	237
E3–E4	297
N1–N2	147
N2–N3	234
N3–N4	224
W1–W2	202
W2–W3	212
W3–W4	409

**Table 3:** The categorization of fish gut fullness level.

Gut Fullness Level	Classification Criteria
0	No food or very little food in fish gut
1	The food volume accounts for 1/4 of fish gut
2	The food volume accounts for 1/2 of fish gut
3	The food volume accounts for 3/4 of fish gut
4	Food fills the whole fish gut
5	Food fills the whole fish gut and makes the gut swell

**Table 4:** Individual size of *Cyprinus chilia* juveniles in Qilu Lake pre- and post-release.

Days Post-Release	Total Length (cm)	Body Weight (g)	Condition Factor (g/cm^3^)	Sample Size
0 (pre-release)	10.8 ± 1.2 ^a^	17.54 ± 5.19 ^a^	1.33 ± 0.09 ^a^	46
1	10.7 ± 1.8 ^ab^	16.58 ± 8.55 ^ab^	1.25 ± 0.15 ^ab^	105
2	9.7 ± 1.4 ^abc^	11.44 ± 5.74 ^abc^	1.18 ± 0.14 ^ab^	90
3	9.3 ± 1.3 ^abc^	10.36 ± 4.37 ^bc^	1.22 ± 0.12 ^ab^	40
4	8.7 ± 1.1 ^c^	8.14 ± 2.96 ^bc^	1.20 ± 0.21 ^ab^	17
5	9.9 ± 2.1 ^abc^	12.70 ± 6.86 ^abc^	1.24 ± 0.17 ^ab^	9
6	9.7 ± 1.7 ^abc^	11.08 ± 6.09 ^abc^	1.13 ± 0.13 ^b^	9
7	9.1 ± 0.4 ^bc^	9.57 ± 1.52 ^bc^	1.27 ± 0.21 ^ab^	6

Note: Means followed by standard deviation. Different superscripts indicate significant difference.

**Table 5:** Size of cultured *Cyprinus chilia* juveniles that moved by direction post-release into Qilu Lake combined for the entire 7 day period.

	Stay in the Release Site	Eastern Lakeshore	Western Lakeshore	Northern Lake Area
Mean total length (cm)	9.5 ± 1.7 ^b^	10.3 ± 1.6 ^a^	9.7 ± 1.2 ^ab^	10.8 ± 2.0 ^a^
Mean body weight (g)	11.39 ± 7.02 ^b^	14.28 ± 7.50 ^a^	11.72 ± 5.04 ^ab^	16.39 ± 8.34 ^a^
Sample size	100	135	31	10

Note: Means followed by standard deviation. Different superscripts indicate significant difference.

**Table 6:** Water depth selectivity of cultured *Cyprinus chilia* juveniles released into Qilu Lake.

Water Depth (cm)	Selectivity						
	Day 1	Day 2	Day 3	Day 4	Day 5	Day 6	Day 7
≤200	0.40	0.78	0.58	0.50	0.54	0.53	0.30
201~300	0.48	0.22	0.42	0.50	0.46	0.47	0.70
301~400	0.12	0	0	0	0	0	0
401~500	0	0	0	0	0	0	0

## Data Availability

All DNA sequences were deposited in the NCBI Sequence Read Archive database with the accession number PRJNA999371.

## References

[1] Zhang C., Xu B., Xue Y., Ren Y. (2022). Fisheries stock enhancement assessment, Progress and prospect. J. Fish. China.

[2] Molony B.W., Lenanton R., Jackson G., Norriss J. (2003). Stock enhancement as a fisheries management tool. Rev. Fish Biol. Fish..

[3] Carrera-García E., Rochard E., Acolas M. (2017). Effects of rearing practice on post-release young-of-the-year behavior: *Acipenser sturio* early life in freshwater. Endanger. Species Res..

[4] Taylor M.D., Laffan S.W., Fairfax A.V., Payne N.L. (2017). Finding their way in the world, Using acoustic telemetry to evaluate relative movement patterns of hatchery-reared fish in the period following release. Fish. Res..

[5] Noda T., Wada T., Iwasaki T., Sato T., Narita K., Matsumoto I., Hori T., Mitamura H., Arai N. (2019). Post-release behaviors and movements of cultured and wild Japanese eels (*Anguilla japonica*) in a shallow brackish water lagoon in northeastern Japan. Environ. Biol. Fish..

[6] Okuyama J., Shishidou H., Hayashibara T. (2019). Post-release horizontal and vertical behavior and philopatry of deepwater longtail red snapper *Etelis coruscans* around a bank. Fish. Sci..

[7] Wu J., Li F., Du H., Zhang H., Wang C., Li J., Wei Q. (2019). Movement of the matured Chinese sucker (*Myxocyprinus asiaticus*) after releasing in the Yangtze River. J. Fish. Sci. China.

[8] Costas N., Álvarez M., Pardo I. (2013). Stocking efficiency and the effects of diet preconditioning on the post-release adaptation of hatchery-reared juveniles of Atlantic salmon (*Salmo salar* L.) in an Atlantic temperate stream. Environ. Biol. Fish..

[9] Hervas S., Kai L., Shane M.A., Drawbridge M.A. (2010). Quantitative assessment of a white seabass (*Atractoscion nobilis*) stock enhancement program in California, Post-release dispersal, growth and survival. Fish. Res..

[10] Whitney N.M., White C.F., Gleiss A.C., Schwieterman G.D., Anderson P., Hueter R.E., Skomal G.B. (2016). A novel method for determining post-release mortality, behavior, and recovery period using acceleration data loggers. Fish. Res..

[11] Munakata A., BjÖrnsson B.T., Jönsson E., Amano M., Ikuta K., Kitamura S., Kurokawa T., Aida K. (2000). Post-release adaptation processes of hatchery-reared honmasu salmon parr. J. Fish Biol..

[12] Howell B.R. (1994). Fitness of hatchery-reared fish for survival in the sea. Aquac. Fish. Manag..

[13] Leber K.M., Brennan N.P., Arce S.M. (1998). Recruitment patterns of juvenile, cultured pacific threadfin, *Polydactylus sexfilis* (Polynemidae), Released along sandy marine shores in Hawaii. Bull. Mar. Sci..

[14] Zhu T., Hu F., Gong J., Du H., Yang D., Wu X., Li X., Sun Y., Liu K., Liang Y. (2022). Initial post-release migration of hatchery-reared *Cyprinus pellegrini* juveniles in the Qilu Lake. J. Fish. Sci. China.

[15] Chu X., Chen Y. (1989). The Fishes of Yunnan, China (Part II Cprinidae).

[16] Zhang S., Zhang Y., Yang C., Peng J. (2014). Study on artificial domestication and breeding of *Cyprinus chilia* in Fuxian Lake. Mod. Agric. Sci. Technol..

[17] Yin M. (1993). Fish Ecology.

[18] Yang J., Chen Y., He Y. (1994). Studies on fish diversity in palteau lakes of the central Yunnan. Chin. Biodivers..

[19] Alcon-Giner C., Caim S., Mitra S., Ketskemety J., Wegmann U., Wain J., Belteki G., Clarke P., Hall L.J. (2017). Optimisation of 16S rRNA gut microbiota profiling of extremely low birth weight infants. BMC Genom..

[20] Wear E.K., Wilbanks E.G., Nelson C.E., Carlson C.A. (2018). Primer selection impacts specific population abundances but not community dynamics in a monthly time-series 16S rRNA gene amplicon analysis of coastal marine bacterioplankton. Environ. Microbiol..

[21] Ni J., Fu C., Huang R., Li Z., Li S., Cao P., Zhong K., Ge M., Gao Y. (2021). Metabolic syndrome cannot mask the changes of faecal microbiota compositions caused by primary hepatocellular carcinoma. Lett. Appl. Microbiol..

[22] Edgar R.C. (2013). UPARSE: Highly accurate OTU sequences from microbial amplicon reads. Nat. Methods.

[23] Wang Q., Garrity G.M., Tiedje J.M., Cole J.R. (2007). Naive Bayesian classifier for rapid assignment of rRNA sequences into the new bacterial taxonomy. Appl. Environ. Microb..

[24] Ferter K., Hartmann K., Kleiven A.R., Moland E., Olsen E.M. (2015). Catch-and-release of Atlantic cod (*Gadus morhua*), post-release behaviour of acoustically pretagged fish in a natural marine environment. Can. J. Fish. Aquat. Sci..

[25] Hoolihan J.P., Luo J., Abascal F.J., Campana S.E., Metrio G.D., Dewar H., Domeier M.L., Howey L.A., Lutcavage M.E., Musyl M.K. (2011). Evaluating post-release behaviour modification in large pelagic fish deployed with pop-up satellite archival tags. J. Mar. Sci..

[26] Wilson A.D.M., Hayden T.A., Vandergoot C.S., Kraus R.T., Dettmers J.M., Cooke S.J., Krueger C.C. (2017). Do intracoelomic telemetry transmitters alter the post-release behaviour of migratory fish?. Ecol. Freshw. Fish.

[27] Watz J. (2019). Structural complexity in the hatchery rearing environment affects activity, resting metabolic rate and post-release behaviour in brown trout *Salmo trutta*. J. Fish Biol..

[28] Ingram T.R., Tannehill J.E., Young S.P. (2013). Post-release survival and behavior of adult shoal bass in the flint river, Georgia. N. Am. J. Fish. Manag..

[29] Wang Z., Chen G., Zeng L. (2018). Study of fish behavior using acoustic fish tags and wireless tracker. South China Fish. Sci..

[30] Larocque S.M., Lake C., Johnson T.B., Fisk A.T. (2022). Patterns in spatial use of land-locked Atlantic salmon (*Salmo salar*) in a large lake. J. Great Lakes Res..

[31] Liu Y., Yang C., Shan B., Sun D., Liu S., Li T., Liu M., Xie Q. (2019). Investigation of a mark-recapture method of black porgy, *Acanthopagrus schlegelii*, in Daya Bay using plastic oval tags. J. Fish. Sci. China.

[32] Alexander J.G., Furgal S.L., Gorsky D., Marsden J.E., Biesinger Z.F., Lantry B.F. (2022). Evaluation of post-stocking dispersal and mortality of juvenile lake trout Salvelinus namaycush in Lake Ontario using acoustic telemetry. J. Great Lakes Res..

[33] Harris C., Brenden T.O., Vandergoot C.S., Faust M.D., Herbst S.J., Krueger C.C. (2021). Tributary use and large-scale movements of grass carp in Lake Erie. J. Great Lakes Res..

[34] Rodríguez E., García-Encina P.A., Stams A.J.M., Maphosa F., Sousa D.Z. (2015). Metaomics approaches to understand and improve wastewater treatment systems. Rev. Environ. Sci. Biotechnol..

[35] Pemberton J.M., Kidd S.P., Schmidt R. (1997). Secreted enzymes of Aeromonas. FEMS Microbiol. Lett..

[36] Qin R., Wang J., Chao C., Yu J., Wang S. (2021). RS5 produced more butyric acid through regulating the microbial community of human gut microbiota. J. Agr. Food Chem..

[37] Molony B.W., Church A.R., Cabassi T. (2014). Tag loss and movement of stocked yearling rainbow trout (*Oncorhynchus mykiss*) in southwestern Australia. N. Z. J. Mar. Freshw. Res..

